# Inhalation and Consumption

**Published:** 1855-07

**Authors:** 


### HALL'S JOURNAL OF HEALTH.

OUR LEGITIMATE SCOPE IS ALMOST BOUNDLESS: FOR WHATEVER BEGETS PLEASURABLE AND HARMLESS FEELINGS, PROMOTES HEALTH; AND WHATEVER INDUCES DISAGREEABLE SENSATIONS, ENGENDERS DISEASE.

VOL. II.] JULY, 1855. [NO. VII.

### INHALATION AND CONSUMPTION.

In the outset we propose some first truths, which had better be scrutinized closely before being admitted, for we wish to wage an open warfare on a fair field. We abominate surprises, ambuscades, and coup-d'etats in all their varieties.

We speak feelingly, for we have been touched in the tenderest of all points—the pocket. But we do not trust our pen to paper in a storm, or at least until we have got used to it: for a man who does that, is very apt to write himself a concentrated ninny. We have been blown up entirely by what we now war against, and a reason of the war is, the diminution of our professional receipts one-half; that, however, is a mere private matter, but "that a generous public should be fleeced first, and then left to die by default, or killed out and out by malpractice, is too bad:" that is to say, *Buncombe* would discourse thus, not we of the Journal, because we advocate the largest liberty. We hold that if people have made money honestly, they have a right to spend it in any manner most pleasing to themselves, provided no wrong is done to another thereby. The world is pretty much made up of humbuggers and humbug-geese (that will do.) But there is a small class of intermediates, very small, you and I, reader, who are neither the one nor the other, outsiders, disinterested spectators, lookers-on in Vienna; and while the Kilkennies are eating each other up—while the riders and the ridden are exhibiting their antipodal feats, we can look on and enjoy the fun.

But as to the first truths:

1st. An abuse of power, of influence, is a great wrong.

2d. An abuse of trust is a greater.

3d. An abuse, abandonment, of principle, is greatest.

If each of these singly is a wrong, then the three combined in one must imply an extraordinary lack of moral rectitude: extraordinary in persons among the common walks of life, but when found among the educated, the elevated, the progressed, we may well pause and inquire, are the foundations of society resting on enduring ground? This is a medical question, but being one in reference to Consumption and kindred diseases, it more or less personally concerns every man, woman and child in the land. The treatment of it by "Inhalation," by drawing into the lungs air impregnated with medicinal substances, has been largely written about, and the propagation of the news of it has been so extensive, that it has not remained unpublished in perhaps any single county in the Union. The persons who practice this treatment are making money by tens of thousands, the rush to their offices, as reported, is extraordinary.

The question is two-fold:

1. Is there purely any such practice?

2. Is there any conclusively reliable evidence of its sole and permanently curative power?

To both these inquiries I answer with a decided "No."

Let it be remembered that "Medicated Inhalation" is the great, the overshadowing treatment insisted on. In reading the publications on the subject, the uninitiated do not dream that anything else is requisite, that any other means are worth a passing thought. "It seems very reasonable," has been repeated a thousand times, "that if the lungs are affected, the most speedy means of cure are to make applications to the affected part directly." This, too, is the published argument, followed up by the assertion, that the old mode of taking things into the stomach, to cure a lung affection, is simply ridiculous.

Can the reader remember any human ailment which is radically cured by only appliances to the spot diseased? We have been in the habit of thinking that the only safe and radical cure of disease is through the general system, is constitutional. If you cut off a weed at the surface, it soon sprouts again, either there or elsewhere; if you take it up by the roots, there is an end of it. So it is with all sickness: it is not by putting on, but putting under, that we can expect to cure. The foundation of disease is in the state of the blood; the blood is made out of the food we eat; if that food is manufactured perfectly, good blood is made, and we get gradually well. The duty of the physician is to superintend that manufactory, to regulate it by means best adapted to each case, whether by food, by medicine, by air and exercise, by water, or by infinitesimal dilutions; but all these are intended to act on the stomach, liver and bowels, these being the great manufactories of the blood.

Now is it not most extraordinary, that a few men within a twelvemonth—men who were never heard of beyond the circuit of a dozen miles of their own homes—should scatter themselves over this whole land, locating in every principal city and town—an Englishman in one place, an Irishman in another, an imitative and enterprising Yankee in a third, while in a fourth a "Hair Dye Vender" hangs his banner to the breeze, according to Rees' Medical Gazette for April, 1855, p. 178. That such a class of persons should suddenly discover that all previous medicine was founded on false principles, and that it was reserved to them to be heralds of the true, in these later ages! these things should at least cause us pause. That the people should swallow them all is not particularly surprising, for that personage can swallow anything, as history abundantly testifies, even itself.

Reader, can we induce you to think for half a minute? You have cut a piece out of your finger before now, or chipped off a bit of skin; it was trifling, you let it alone, and it got well, it healed up. Could any external appliance known to man have replaced that bit of flesh or skin? yet it was replaced. How? The blood bore there the materials of repair, which materials were drawn from the stomach, from the food you had swallowed. And yet, without a moment's reflection, you pronounced it "very reasonable that the lungs should be healed by appliances made directly to them, instead of the stomach," and you posted off to get an inhaling apparatus, and have been puffing away for very life ever since at thirty dollars a month, a dollar a day in advance; why it is worth that to "blow" all day; a good many people make a living by doing that and nothing else, while you pay for the privilege. Alas for Jonathan! he must be in the sear and yellow leaf, for he is losing the cuteness on which he has prided himself so long. Think again, bilious and other fevers have been cured millions of times. Bilious fever and jaundice are founded in the liver. Medicine is swallowed into the stomach, and the man is cured. Has any one ever been so insane as to propose to cure bilious fever or jaundice by making applications directly to the liver? You have had a violent headache, have eaten a good portion of a fine turkey, aided and abetted by a glass or two of champagne; and by the time the turkey and champagne were gone, the headache was gone too. Is it likely the headache would have disappeared had you poulticed the skull with a conglomeration of wine, turkey, and its etccteras?

So far from its being "*reasonable*" that a malady should be more speedily cured by applications made directly to the spot, it is in many instances the most certain means of destroying life. Every educated physician knows that many a life has been lost by applications to old sores, which caused them to heal up there, only to be manifested in some more critical part. How many have lost life by skin diseases being "*driven in*" by external appliances, as in hives, measles, chicken pox? A case occurred not long ago, where a man was advised for a scratch on the breast to rub it with candle-grease; he did so, and died in consequence, some verdegris having been formed on the candlestick. One of our prominent citizens was advised to put a little creosote on a pimple on the shoulder of his little child; he did so, and the child died in convulsions within twenty-four hours. Multitudes of instances can be given, showing that local applications are attended with serious consequences: showing that they are exceedingly dangerous, and that NATURE CURES NOTHING IN THAT WAY, not even the scratch of a pin or the gash of a knife, but that her cures are through the roundabout way of the general constitution. Instances are numberless in our reading or observation, where men and animals from accident or design or war, have been left in the open field for dead, so cut and gashed and bruised were they; and yet afterwards revived, and crawled to some shelter and got well, without any medical means whatever, internal or external, nature applying nothing to the wounds, but gradually supplying them with reparative matter through the blood. So that the most palpable of all truths is, that *applications to the spot diseased is never natural*, it is directly the reverse of nature's mode. We may well be suspicious of the truth of any system, the champions of which resort to means of support which are roundabout, to give it no harsher name.

What is the *modus operandi* of these advertising men, these advocates of inhalation ? Not wishing to be personal, we will use fictitious names. An advertisement is before me, headed "Inhalation," stating that "Dr. Jean, one of the most celebrated physicians in New York, writes as follows," (which is omitted) and signs his name, street and number; on turning to the Directory, we find no such name in it, nor on the door of the house specified. Appended to the letter is the "CAUTION," that this vapor is "the original and only genuine article."

Another Inhalist is at the expense of advertising the public extensively, and especially strangers who come to New York, that wishing them not to be imposed upon by pretenders, he takes this means of advising them to come to him, and thus not fall into the hands of imposters.

In the Daily Times of Saturday, June 2d, 1855, appears the following, names omitted:

[Advertisement.]

**Consumption, and How it May be Prevented.**

Dr. ——'s letter, which should appear in our columns of this morning, is necessarily deferred until Tuesday next. It will be found to be the most *truthful* and *powerful* article ever written upon the *proper* and *only* method of *preventing* Consumption. It embodies more *sound, practical common sense* upon this subject than has been published by any physician for the last hundred years. He tells the consumptive invalid "the truth, the whole truth, and nothing but the truth." Dr. ———, who has charge of the ———, is justly considered to be the most *correct and accurate* detector "of the various diseases of the lungs" and throat in this country. We would advise those of our readers who have perused his previous articles to read with attention the one which will appear on Tuesday next.

The mass of readers not noticing the word "*Advertisement*" in the smallest letters in the article, would suppose it was written by the editor of the paper. It certainly reads as if the editor had written the notice. The impression made is certainly that some other person than the Inhalist was the writer. All of us know, however, that it was written under the direction of the man himself. If Inhalation be true, can it require a course like this? And here is a newspaper, willing to be a partaker in all this, for two dollars. See our three first truths.

Another of these men writes:

"Who is Cock Robin?"

and goes on to say who he is, where he was born, how he came to be possessed of the great health secret, the long years he spent in studying it out, his wonderful success, and seeing that success, he considers it his duty to take this method of letting a suffering world know it. He carries it to a publisher, whom he has taken pains to ascertain beforehand, will do anything for pay, lays down five hundred or a thousand dollars, next day the paper appears with the aforesaid Autobiography, "We hasten to present to our readers the following interesting autobiography of an eminent physician." If there is still some little conscience for truth left, it is simply headed, "For the Daily Bribee," or more modestly still, "Communicated." Now and then we see a paper too independent to be so palpably *particeps criminis* of deliberately deceiving its patrons, and gives the truthful heading, "Advertisement," then its intelligent readers know where they stand; still, the masses are not intelligent, and are consequently deceived, hence again the violation of our *First Truths.*

But this is not all; the next step is to purchase extra copies, these are sent off to the country newspapers, with a letter inclosing five, fifty, or a hundred dollars, with a request for insertion *among the reading matter* as credited to the *Daily Bribee*, or other paper supposed to have a wide name, reputation and influence, as if the article had found its way there by virtue of its inherent importance.

This is the operation, and in view of it, does Truth require such tortuous modes of propagation? If so, we say let such truths perish!

There are a few newspapers, which scorn to participate, even remotely, in trickeries like these. The religious press, strange to say, is not among these honorable exceptions, as a class, edited, although they be, by clergymen, whose name and office and standing are thus prostituted to the dissemination of these deceptions among their wide list of patrons, who look up to them for all that is truthful, and open, and fair.

I wish in the most direct manner to offer the explanation of *facts*, in behalf of conduct so apparently inconsistent on the part of men, to whose talents, and education, and piety, and good citizenship, this great land owes, to a principal extent, the existence and the perpetuation of its liberties. Clerical editors are not the owners of the papers they conduct, they are merely paid to provide suitable *reading* matter; all *paid* matter is under the sole control of the owner of the paper. Still, the power which every editor has over his readers, his influence, is on the side of the deception, for the great mass of readers, out of cities, take it for granted, that impositions would not be allowed by a clergyman; they hold him to a certain extent responsible for all that appears in his paper, advertisements and all. This is wrong to be sure, but an editor knows this feeling does exist, and knowing it, is to a degree responsible. There is not a religious newspaper in the land, perhaps, which would not feel itself insulted, if a liquor dealer were to offer an advertisement of his wares; and yet, these very papers, with some exceptions, advertise patent medicines without any compunctions whatever. They write ravingly against men whom they suspicion of selling adulterated, drugged liquors, and yet they will advertise drugged molasses and water, and be glad of the job, because all patent medicine dealers "*pay well.*" But public opinion is *against* drugged brandy, and *for* drugged molasses and water. That's the reason, because why!!!

In this manner is the clergyman used as the cat's paw of the mercenary publishers, in favor of quackery against regular medicine, to which his own intelligence compels this same clergyman to resort, in case of illness; and yet, with the expectation of gratuitous services. Such expectation, be it known, is not realized in us.

This detail of facts, may well originate the inquiry, Ought a clergyman to accept the editorship of a religious newspaper, without the provision that nothing unbusiness-like or deceptious, should ever appear in any department of the paper? As it is, our *First Truths* are all violated in this thing, practically violated by the best educated, the most refined, and progressed men in the land.

There are instructive side inquiries, which may throw some light on *Medicated Inhalation.* If it is so efficient a remedy, why should these men have left their native land, where the disease more abounds than here? Or, if leaving for any necessary reason, why was not some agent, or friend, or relation left in London, or Paris, or Berlin, to afford their own countrymen the full benefits of this great discovery?

If the system possesses substantial merit, why is it necessary to keep it before the people, at the expense of tens of thousands of dollars, and at the greater expense, to an honorable mind, of personal respect, which is involved in the advertising practices already referred to? We would suppose that every person cured, would be a walking advertisement, not up and down Broadway, but all over this broad land, until soon, there would not be a cow path in all America untravelled by them, and no advertisement would be needed.

If "Inhalation" is the great remedy for Consumption, why is it that cart loads of medicines are wheeled from the doors of these men, and white powders, and morphine, and other anodynes are nightly swallowed by their direction to abate the cough, or has every patient a cough in the stomach? Or if so, why do these men reiterate, day after day, in the public prints, "that the swallowing of drugs and medicines is the discovery and invention of human minds and means, against the indications of nature and common sense?" This is the verbatim language, the literal conduct being to send medicines by mail and express to swallow nightly. Can truth stand in need of such aids? Would truth deign to countenance them?

It is a broad truth among men, that a gentleman, among gentlemen, is the last one to imagine a dishonorable act on the part of his compeers, but even if imagined, he would sooner make the largest sacrifices, than frame his suspicions into words. Yet one of these Inhalists publishes to the world that he *charitably* believes that the *great majority* of physicians know the inefficacy of drugs and medicines, but employ them still, rather than be *candid* and *honest* to their patients, and yet these same persons send medicines to swallow nightly, in one case I know.

If Inhalation is the remedy, why is it that in some cases patients are visited daily, and as often as one medicine fails, another is substituted, until the mantel is filled with vials, and packages, and all that ?

If Inhalation is the remedy, why is it that some patients who have cough are not advised to use it, and why is it, too, that after four, and five, and six months' diligent attendance, persons have left not a whit better than on the first day of trial, and others go steadily down to the grave, whose certificates are not asked or wanted ?

If Inhalation is the great remedy, why is it that in every section of the country, men are found practicing it, each claiming for himself the originality, and branding the other as an "imposter," "pretender," "shark," while a third party gets up an "*improved*" *inhalation;* and why is it, that each one claims for himself to have the *only* curative inhalent, while another, who sends out annual circulars by the scores of thousands, has *within a year* paid hundreds of dollars for a single advertisement, in which medicated inhalation is not even hinted at, *now* publicly announces that he has used it among other means for years, and heads his advertisement with *Inhaling Treatment*, as if it were the principal means of cure, followed by certificates of cure, which say not one single word about *Inhalation.* Showing that these men watch the wind, and sail with it. They go with the tide. They ride triumphantly on popular credulity, the people paying for being thus ridden, which they have a right to do.

One of the New York dailies in a recent editorial, takes upon itself to say that the "letters" published so extensively on medicated inhalation, are "at once clear, learned and free from charlatanism," yielding to the author "an income of over a hundred thousand dollars a year," and that "there is no reason to suppose him to have been actuated by a desire to make money." And commends the "*manly boldness*" of one who "breaks through the conventionalities of his profession." There is honor among even thieves, except those who are below them. These are a law unto themselves. The editor commends this breaking away from professional conventionality. It pays him well to do so, the paper having, on its own statement, received eight hundred dollars for one such job. Would that same paper break through the professional conventionality of the Printers' Trades Union? Would not the journeyman printer, who had once given his word to be regulated by a certain standard of prices, be looked upon with loathing by his fellow "jours" the moment they found him recreant to his obligations, more sacred than an oath, the only bond being *pledged honor?* Such is the bond among honorable and regularly educated physicians, and yet this paper speaks of these violations as an act of *manly boldness* at least in another, but incapable of such an act itself, that is, until "*its price*" is offered. We might well inquire, how this sub-editor knows that these "letters" are *clear, learned and free from charlatanism?* To be be capable of such a criticism, the whole range of medical science should be familiar to him, while the expression itself bears ample evidence of his ignorance and presumptuousness in pronouncing that "*learned*" which is a tissue of ingenious misrepresentations, and in dictating to the medical profession what should be its conventionalities; and in further stating the monstrous untruth, that it is *dangerous to professional standing to announce in a respectable journal any new remedy discovered or any new operation hit upon.* So far from such being the case, there are scores of journals published in the United States for the express purpose of communicating new truths and discoveries. No sooner does a regular physician become convinced of an important new practical truth or remedy, than it is sent to one of these journals and within a year is known, *with all its minutia*, to the medical profession of all civilized nations. This is a universal conventionality, and it is the dereliction of so humane, so unselfish, and so magnanimous a regulation, which consigns all these advertisers, in the estimation of educated physicians, to a position below entitlement to the States prison, because it is humanity, education and oath of honor, all violated at one fell swoop; for each inhalist proclaims his own as possessing the distinctive curative quality, but what that is, he locks up in his own bosom; and this is what the paper before us characterizes as *manly boldness.* "Boldness" indeed it is, but as to its manliness, that is another question.

When a man once steps aside from an honorable path, when he once violates his convictions of truth, when he once descends to trickery, no *optics sharp* can see where that man will go, no divining rod can measure the depth of degradation to which he may descend. Really we are waxing warm, and perhaps had better sail on another tack awhile, lest we should become personal. We will finish the sheet and cool off at Barnum's baby show. But we are almost unrestrainably inclined to give a medical cotemporary a dig, for lending its columns to the purposes of these advertising inhalists. I was surprised. Physicians in New York who are honorably and proudly known everywhere, and who have been here much longer than I, have stated that "*it did not surprise*" them. By which we learn the fact, "that the same things affect different persons differently." *Quod erat demonstrandum*, as Euclid would say.

One other fact worth noting is, that some of these inhaling men, have for ten and twenty years been treating consumption as a speciality, and yet are changing their treatment, from time to time. Within fifteen years, the Inhaling Tube was furnished to every patient, as *the* thing essential to a cure in every case. In process of time, these same men laid aside the tube, when it became an old thing, and a common quill answered the purpose, without the expense of a five dollar tube, which cost fifty cents, and the *Abomable Purporter*, as dame Partington would say, became the rage. Braces were to cure every thing. If a man had throat disease he had only to have something to hold up his belly. If his lungs were affected, the same thing was advised. If he was of the slab-sided sort and had no abdominal, the supporter was still advised to be applied, until the patient could "*get up*" one, as a supporter was certain to improve digestion, which was the first step towards getting fat. The advocate of this theory announced publicly that he had a great many cases, amounting to thousands, notes of which had been carefully and industriously kept, amounting to "ninety volumes," which, "in the language of the London Lancet, forms one of the most valuable and comprehensive body of notes ever presented to the public eye." Now, a casual reader would suppose that the London Lancet, which is perhaps the leading monthly medical publication in the world, had made this statement in reference to these very "ninety volumes" of notes made by the advertiser, when in reality, they were made in reference to the private notes or medical cases of an eminent London practitioner, who had just died. Whether it was the design of this person to produce the impression, without saying so, in as many words, that this quotation was made in reference to his notes, the reader must judge for himself. But the question again recurs here, Can truth need such aids as these?

But notwithstanding "ninety volumes" of such notes were made, "which, in the language of the London Lancet, forms one of the most valuable and comprehensive body of notes ever presented to the public eye," and notwithstanding these notes were avowedly made "in treating forty thousand consumptive persons, nineteen out of twenty of whom recovered," without even mentioning medicated inhalation, but by relying on the tube first, and then the "Purporters" aforesaid, this same person, within a year of such advertisement, now comes before the public with "Inhaling Vapor" at the head of his advertisement, affirming, in addition, that he has been using medicated inhalations for years, and that he is therefore not a new man in these things, like the others are.

But what, after all, is the succinct history of Inhalation as a means of cure for pulmonary affections? Simply this, it was proposed a half a century ago. Sir Charles Scudamore wrote and wrecked himself on the practice; and every few years since, it "*turns up*" again like sharp-toed shoes, or trowserloons, with suspenders at both ends. But each successive *Redivivor* assures the public, that the reason of previous failures was that the genuine inhalent was not used until he, by some rare chance, discovered it. Yet he fails to let the public know what his inhalent is, so that they might know whether it was different from all others or not.

Inhalations of air saturated with moisture or medicants, have been used by the regular profession for hundreds of years in cases of affections of the air-passages—☞ *but only as temporary alleviants, to gain time for the employment of more efficient means*, and this is all they are worth. But to stifle coughs and disease by the fumes of alcohol or opium, these being the principal things which these men use, though they affect to put in something else of more primary importance, as a blind, is no more curative than shutting down the hatches of a vessel, whose hold is on fire; only retarding for a season, to break out with greater malignity. That some men, having a slight cough of long standing, or a severer form of it of more recent date, do get well under medicated inhalation, is no proper proof, for millions get well of both, while doing nothing.

The fact is, time has not been allowed to test the reality of a single cure, for it is less than two years since we saw the first certificate of cure, and it is well known that the average duration of life even after a person has become consumptive, is near two years.

But by making strenuous efforts to persuade the public that every cough a man has, or every clearing of the throat, or every speck or streak of blood in the expectoration, is a serious sign of consumption begun, whereas a streak or speck of blood in the expectoration is no sign of consumption at all, but is from the upper part of the air tubes or throat, and not from the lungs, but making this impression, and driving the people to them, through fear on the one hand, and promises of certain cure, by easy means, on the other, it is no wonder that their receipts are counted by tens of thousands of dollars; for such ailments would in many instances disappear of themselves, with slight attention to diet, and bowels, and exercise; but, under the circumstances, inhalation gets the credit of cure in an ignorant mind, each of which is a walking advertisement, a peripatetic blower, or a drummer up, for the Inhalist.

Let every reader propose to himself this simple test-question :—

Do I know a single individual who had serious symptoms of actual consumptive disease of several months' standing, who a year ago placed himself under medicated inhalation, and is now a well man?

I will pledge my existence that not one single such case can be found. I defy every Inhalist in the Union to produce from their combined practice one well-marked case of this kind. And more, no man of observant intelligence, and of what we call "position," can be produced, who will affirm to any such statement in his own experience.

If there had been curative merit in the practice of inhalation as to efficiency and permanency, physicians of undoubted standing and of educational ability, would have long ago instituted a searching investigation, but as far as I know, not one single such physician has thought it worth while to investigate so transparent a fraud; for in medicine, fraud and secrecy are twin sisters, and each of the hundreds who practice inhalation, claim to be the possessor of "THE" secret, *and keeps it.*

*The practice of all honorable physicians, be it known, is, over the world, one and the same: the very moment they are satisfied of having discovered a new and valuable remedy, it is sent off to some respectable medical periodical, and within a year, the remedy and its uses, is in the possession of every educated physician in Christendom. It is considered a mutual duty, and he who fails of it, is immediately placed beneath contempt.*

If there is any error on this subject, it consists in making premature publications. The anxiety to communicate what is supposed to be new or valuable is such, that in many instances, time is not allowed to perfect observations, but hints are thrown out, so that the attention of other practitioners may be directed to the same points, thus securing the co-operation of many observant minds towards one point, by which the public are every way benefited, while the discoverer thus voluntarily loses the chance, as it were, of securing a higher distinction and a greater glory by perfecting the discovery within himself. If any set of men on the face of the earth can give equal proof of a pure magnanimity to those constantly given, as a habitual thing, as a matter of course, as a sense of professional courtesy and duty, I have yet to know it. And these are the men to whom interested and paid editors volunteer advice designed to throw unnumbered thousands of money into their own pockets, as to what is conventional propriety and morality—these are the men whom advertising inhalists charge with "a lack of candor and honesty" in administering drugs which they know are useless, while these same inhalists conceal the means of cure, which, if what they say is true, would save multitudes from a consumptive's grave; and yet, rather than give the profession the knowledge of their secret, they keep it to themselves, and allow all to die who cannot receive the boon at their hands. Well may it flush the cheek of a man, to think that such can claim the name of human.

These are the men whom the press of this country laud as exhibiting "manly boldness, in breaking away from professional conventionalities," helping them, by *good editorials*, well paid for, to get rich at the expense of the health and happiness of their patrons. Gentlemen of the tripod, we ask you to make a note here. *Et Tu, Brute!* are you also, with your superior education and habits of thought, are you also actuated by the same seven principles of the vulgar multitude away down yonder, to wit, the five loaves and the two fishes, five and two making seven? *Proh Pudor!*

But the Public is a free horse, and as long as it likes to be ridden, let it please itself. Why, then, do we write upon this subject? To regain the hundred per cent. of receipts which we stated to have lost in the commencement of this article? Not at all, for in the reaction, our receipts from professional sources are larger now, and have been for some months, than they have been for corresponding months since we came to New York, without any unusual efforts on our part. We write more for future capital than for present profit, hoping, however, that we will be the means of imparting useful information to the observant few, and thus contribute something towards building that great temple of truth which is to make this earth a paradise for man now and a heaven hereafter.

From all great public delusions, great practical public truths are learned, and so in this particular case. When the man who "cured nineteen cases of consumption out of every twenty," by using the inhaling tube and the abdominal supporter and shoulder brace, and while doing so made ninety volumes of notes, which, "in the language of the London Lancet, forms one of the most valuable and comprehensive body of notes ever offered to public inspection"—when these are suddenly dropped as principal agents, and medicated inhalation is placed in glaring capitals at the head of the advertisements, what can we suppose other than that the tube and the supporters were ridden as hobbies to make money, and when the hobby would not ride any longer, the first fresh nag that came along was appropriated, and the Gilpin race began anew.

It may be instructive for me to state here, that I think I was the first regularly educated physician in the United States who began the treatment only of consumptive diseases, and I continue, as far as I know, to be the only physician in the United States, who rigidly confines himself to the treatment of such diseases, except when called upon by friends or neighbors in emergencies, when no charge is made.

Such being the facts, it is natural that I should be better posted up in what pertains to Consumption, than perhaps any other. And not being dependent on my practice for a living, I have not the inducements which may beset others to miscolor or distort in any direction. I think that no man can be happily and permanently benefited by laboring to produce an untruthful impression under any conceivable circumstances; on the contrary, they who closely practice the truth will live and prosper by it. Really the Baby-show has had a wonderful effect. Awhile ago, we had "*waxed warm*," went to Barnum's our Rib along, and lo and behold, we have become ethical and homiletic. We think of hinting to Barnum the propriety of making the Baby-show a permanent institution of the country. We think it would be a good idea to make a baby-show an accompaniment of the next Congress of Nations for the promotion of peace principles, just as the jewsharp, the fiddle, and the banjo are accompaniments of a monkey-show—they help to harmonize. Who can feel warlike after looking at *the tweetest itty bitty baby ever was, to tweet can't be any tweeter*, and if the sight of *one* baby has such a mellifluent effect, what would be the effect of seeing the hundred and forty which Barnum got!

We suggest that Barnum and his Babies be appointed Uncle Sam's representative to the next Universal Peace Congress. By the way, Barnum ought to divide a portion of the profits of the show with the Journal, the first hint of such a thing having been given in one of our earliest numbers. In fact, we beat Barnum. Just as Fowler told us not long ago, when we made an experiment on him *in cog.* He made our *originative* bump one of the largest. But hoid—may-be he meant we were fabricative, in the moral sense! Never thought of that until this moment. The fact is, we never had any respect for phrenology; its *dictas* may be interpreted to mean every thing or nothing. Besides, some fifteen years ago he told us we were good at any thing, great in nothing; so we left in disgust, and now that he tells us we are great at fibbery, we are "disguster."

My own views are, that no medicine known has any direct curative effect in consumptive diseases; that the only cure is in the perfect action of the *Chylopoietic Viscera*, that is, of the food digesting and blood making organs, the chief of which are the stomach, liver and bowels.

Consumption is literally *defective nutrition:* the whole man dwindles; flesh, strength, breath, all waste away by painfully slow degrees; *not the lungs alone*, which make but a small part of what is really affected, the whole machinery of the man is in disorder. The patient may eat heartily to the last day almost, yet the system has not the power to extract from the food eaten, and properly appropriate, the nutriment which it naturally possesses; hence the uniform remark "I eat well, but it does not seem to strengthen me." In almost all other diseases, the ability and appetite to eat is followed very shortly with increasing flesh and bodily vigor. How then can medicinal substances applied to the lungs, have any really curative effect, when it is in the stomach, liver and bowels, where the very foundation of the ailment lies? for it is there the blood is made, out of which the tubercle, the seed of consumption is formed, as all allow. The blood is not made consumptive in the lungs, but carries consumption with it to the lungs in its imperfect elimination, in its imperfectness of material, which material is drawn from the food after the stomach has acted upon it. And all that medicine can do towards curing consumption, is by the aid which it affords the stomach, liver and bowels in calling for, in receiving and in digesting the food which we swallow. Whatever gives appetite, and with it an increase of digestive power, that is radically curative of consumption. Any man who can be cured at all, will be cured by taking into his lungs the largest amount of out door air, and by imposing upon his body the largest amount of muscular exercise, not involving actual fatigue, the most favorable combination of all which, is found in continuous, active, horseback exercise on the open road, from morning till night, from one week's end to another, with some pleasurable and profitably absorbing object ahead, other than the mere health; in all of which, being under the care of some *one*, honorable, educated physician who possesses your confidence and respect. This, reader, is the embodied result, without any mental reservation whatever, of the observation and experience of a young life time, spent in the treatment and study of this one disease, and upon its truth, I, with steady confidence, do stake my reputation and my daily bread. But why is active exercise on horseback on the open road so largely insisted on? simply because in the experience of all, nothing else is so effectual in giving a good appetite, and securing a good digestion; the exercise giving the digestion without the over fatigue attendant on bodily labor, and every breath drawn being a breath of pure air, unloads the blood of its impurities in the lungs, to be sent thence to the most distant portion of the human body at the rate of a hogshead an hour; imparting life and energy to every fibre.

What then the need of a physician? To know *first* the actual condition of things and to meet incidental and occasional symptoms promptly, before they gain power. An infant's arm may stop an avalanche, a moment later and millions of men are powerless. And then again, comparatively few have it in their power to carry out the practice indicated, at least to its full extent; then, the judgment, tact and experience of the physician are to be brought into requisition to provide substitutes, medicinal and otherwise.

It is not contended that there are no benefits whatever to be derived from breathing air saturated with something else, whether drugs, liquors, aliments or other things, but we do contend that such effects are transient, are not radically curative, but mere alleviants, only giving time for other more efficient means to be brought to bear. We tried the whole thing years ago and found no worthy result in any single instance. But say these new men, "that was because you did not use the right material and in a proper manner, but we have found out a new inhalent, and hence our success." How do the people know that these men use a new inhalent, as long as they studiously conceal their secret? If it be really new, why is not the name and all the minutiæ given? If it be a remedy of striking efficacy, the State of New York alone would give them millions of dollars every year, beyond what they can ever use, while millions of their fellow creatures outside of New York would be saved if the instrumentalities were committed to other hands. The Arctic is going down, one man alone of all the throng knows the means of safety, and could in a moment's time impart instructions which would enable others to be as efficient in saving human life as he himself; but no, he only saves those who place themselves in his hands; the others may perish for what he cares, and yet such men as these are they whom "an independent and intelligent press" are lauding to the skies as men of "*manly boldness*," as men "free from charlatanism and not influenced by mercenary considerations!"

Make the contrast between these advertisers and Dr. Horace Green. When he conceived himself to have become the possessor of one of the most important medical instrumentalities of modern times, he published a book with the fullest details, and opened his office, free of charge, to every man who even said he was a physician, and patiently instructed them day after day, until they could easily perform the operation, when he said to them, "go, cure as many as you can, there is room enough for us all." Can humanity any where show a larger heart than this? and yet such a man, with the whole class to which he belongs, and of which he is a representative, these advertising inhaler's declare, "are not disposed to be honest and candid with their patients, and give counsel upon a subject which they know little or nothing of, neglecting the plain indications of nature and common sense." And in using such language, are complimented by some editors as acting with "*manly boldness*." If this is not a prostitution of power, of confidence, of principle, then is there no such thing under the sun. And if such prostitutions are accomplished in the person of those who are educated, elevated, progressed, well may we contemplate with sadness the fact that we have fallen on evil times.

